# Natural products from plants and microorganisms: Novel therapeutics for chronic kidney disease *via* gut microbiota regulation

**DOI:** 10.3389/fphar.2022.1068613

**Published:** 2023-01-17

**Authors:** Lin Zheng, Mingjing Luo, Haokui Zhou, Jianping Chen

**Affiliations:** ^1^ Shenzhen Key Laboratory of Hospital Chinese Medicine Preparation, Shenzhen Traditional Chinese Medicine Hospital, The Fourth Clinical Medical College of Guangzhou University of Chinese Medicine, Shenzhen, China; ^2^ Shenzhen Institute of Synthetic Biology, Shenzhen Institutes of Advanced Technology, Chinese Academy of Sciences, Shenzhen, China; ^3^ CAS Key Laboratory of Quantitative Engineering Biology, Shenzhen Institutes of Advanced Technology, Shenzhen, China

**Keywords:** chronic kidney disease, gut microbiota, polysaccharide, herbal medicines, phytochemicals, gut–kidney axis, natural products

## Abstract

Dysbiosis of gut microbiota plays a fundamental role in the pathogenesis and development of chronic kidney disease (CKD) and its complications. Natural products from plants and microorganisms can achieve recognizable improvement in renal function and serve as an alternative treatment for chronic kidney disease patients with a long history, yet less is known on its beneficial effects on kidney injury by targeting the intestinal microbiota. In this review, we summarize studies on the effects of natural products from plants and microorganisms, including herbal medicines and their bioactive extracts, polysaccharides from plants and microorganisms, and phytochemicals, on the prevention and treatment of chronic kidney disease through targeting gut microflora. We describe the strategies of these anti-CKD effects in animal experiments including remodulation of gut microbiota structure, reduction of uremic toxins, enhancement of short-chain fatty acid (SCFA) production, regulation of intestinal inflammatory signaling, and improvement in intestinal integrity. Meanwhile, the clinical trials of different natural products in chronic kidney disease clinical practice were also analyzed and discussed. These provide information to enable a better understanding of the renoprotective effects of these effective natural products from plants and microorganisms in the treatment of chronic kidney disease. Finally, we propose the steps to prove the causal role of the intestinal microflora in the treatment of chronic kidney disease by natural products from plants and microorganisms. We also assess the future perspective that natural active products from plants and microorganisms can beneficially delay the onset and progression of kidney disease by targeting the gut flora and highlight the remaining challenges in this area. With the continuous deepening of studies in recent years, it has been proved that gut microbiota is a potential target of natural active products derived from plants and microorganisms for chronic kidney disease treatment. Fully understanding the functions and mechanisms of gut microbiota in these natural active products from plants and microorganisms is conducive to their application as an alternative therapeutic in the treatment of chronic kidney disease.

## 1 Introduction

Chronic kidney disease (CKD) has become an increasing public health problem with a wide range of complications and high risk of mortality. The worldwide prevalence of CKD (8%–16%) brings a heavy economic burden for middle- and low-income countries in particular (Zhang et al., 2012; Lv and Zhang, 2019). Due to the concealed symptom of early CKD, clinical diagnosis and therapeutic interventions for early CKD patients are lagging (Flagg, 2018); most therapeutic methods for ESRD (end-stage renal disease) patients such as lowering blood pressure, controlling blood glucose, and reducing proteinuria have poor effect in preventing kidney failure (Chen T. K. et al., 2019). As current anti-CKD therapies have limited effectiveness and/or severe adverse effects, alternative treatments like natural products have gained much more attention, especially with current studies implying that gut microbiota is an anti-CKD target.

Gut microbiota are composed of over 1,000 genera of bacteria colonized in the human intestine, which play a crucial role in many important human physiological functions, including maintenance of energy balance, modulation of intestinal homeostasis, and regulation of the immune system (Fan and Pedersen, 2021). The features and functional effect of intestinal flora have been extensively studied in the past few decades, and lots of studies have revealed that dysbiosis of gut microflora is highly associated with the onset and progression of CKD (Jiang et al., 2017; Hu J. et al., 2020; Andrianova et al., 2020; Wang X. et al., 2020; Zhong et al., 2020; Du et al., 2021a). Gut microflora influence the gut–kidney axis, namely, the crosstalk between the intestinal microflora, CKD, and changes in the intestinal environment (Evenepoel et al., 2017), in a bidirectional way; on the one hand, kidney function failure is highly related to the reduced bacterial diversity and biased community constitutions of the gut microflora (Cigarran Guldris et al., 2017; Hobby et al., 2019). Increasing sources of evidence support that altered gut microbiota composition has been reported in patients with different kidney diseases, including those with end-stage renal disease (ESRD), acute kidney injury (AKI), IgA nephropathy, CKD, and diabetic nephropathy (Jiang et al., 2017; Hu J. et al., 2020; Andrianova et al., 2020; Zhong et al., 2020; Du et al., 2021a). CKD patients had lower bacterial diversities than healthy subjects, with an increasing abundance of harmful microbes such as ***Proteobacteria* and *Actinobacteria*** and a decreasing level of beneficial microbes such as Lactobacillaceae (Hu J. et al., 2020; Andrianova et al., 2020; Zhong et al., 2020). Moreover, the change in gut microflora structure and function is highly associated with the severity of CKD (Hu X. et al., 2020). Specific gut microbes producing short-chain fatty acids (SCFAs) are potentially helpful for CKD early diagnosis and prognosis monitoring (Gao B. et al., 2021; Sato et al., 2021). On the other hand, various metabolic pathways including uremic toxin, SCFAs, and bile acid pathway are affected by the altered gut microflora. Experimental and clinical evidence demonstrated that altered gut microflora in CKD patients could accelerate the biosynthesis of uremic toxins, whose concentrations would consequently increase in the blood (Wang X. et al., 2020; Rysz et al., 2021). Also, these resulted in the progression of impairment in the intestinal epithelial barrier and inflammation which intensified the cardiovascular and kidney diseases (Espi et al., 2020; Opdebeeck et al., 2020), suggesting that the gut flora could serve as a novel therapeutic target for CKD and related complications.

In order to clarify the gut microbiota and anti-CKD molecular mechanisms of these natural products, we reviewed the knowledge based on publications in English- and Chinese-language journals. Table 1 summarizes the mechanisms of different natural products from plants and microorganisms, including herbal decoction, crude extracts, polysaccharides from plants and microorganisms, and phytochemicals, in CKD progression through targeting the gut microbial based on these studies, which focus on remodulation of gut microbiota structure, reduction of uremic toxins, enhancement of SCFA production, regulation of intestinal inflammatory signaling, and improvement in intestinal integrity of natural products commonly used in kidney diseases. Meanwhile, the clinical trials of different natural products in CKD clinical practice were also analyzed and discussed (Table 2).

**TABLE 1:** Mechanisms of gut microbiota as an alternative target for natural products from plants and microorganisms for the treatment of CKD in animal experiments.

Intervention type	Intervention content	Experiment model	Experiment result	Mechanism					Reference
				Remodulation of the gut microbiota structure	Reduction of uremic toxins	Increase in SCFA metabolism	Regulation of intestinal inflammatory signaling	Improvement in intestinal integrity	
Decoction	Shenyan Kangfu tablet	Diabetic nephropathy rats	Reduced stimulated blood glucose and HbA1c levels, alleviated renal dysfunction, glomerular and tubular damage, and renal inflammation (TNF-α and IL-1β)	√					Chen et al. (2021)


Decoction	Gum acacia	Adenine-induced CRF rats	Restored the depleted butyrate level and various perturbated functional pathways	√		√			Al-Asmakh et al. (2020) and Lakshmanan et al. (2021)
Decoction	Jian-Pi-Yi-Shen (JPYS) formula	5/6 nephrectomized rats	Restored the blood reticulocyte counting and serum calcium level; identified the distinct gut microbiota responses to JPYS	√					Zheng et al. (2020)
Decoction	Huangkui Siwu Formula	5/6 nephrectomized rats	Inhibited the oxidative pathway of tyrosine and decreased the decomposition of PHA, thereby inhibiting PC production; inhibited the conversion of PC to PCS in the liver by significantly downregulating *SULT1A1* gene transcription and protein expression levels		√				Lu et al. (2020) and Lu et al. (2021a)
	Huangkui capsule	5/6 nephrectomized rats	Inhibited indole production of gut bacteria by interfering with tryptophan transportation		√				Wang et al. (2019)
Decoction	Rhubarb enema	5/6 nephrectomized rats	Decreased serum levels of IS, renal oxidative stress, and NF-κB levels		√				Lu et al. (2015)
	Rhubarb enema	5/6 nephrectomized rats	Reduced the serum TMAO and TMA levels; inhibited the expression of IL-6, TNF-α, and IFN-γ; and alleviated tubular atrophy, monocyte infiltration, and interstitial fibrosis		√				Ji et al. (2021)
	Rhubarb enema	5/6 nephrectomized rats	Improved the intestinal barrier, regulated gut microbiota dysbiosis, suppressed systemic inflammation, and alleviated renal fibrosis	√				√	Ji et al. (2020)
Decoction	Zhen Wu Tang	IgA nephropathy rats	Ameliorated microbial dysbiosis and attenuated the renal damage; modulated the metabolic phenotype perturbation	√					Li et al. (2021)
Decoction	*Rheum palmatum*–*Salvia miltiorrhiza*	5/6 nephrectomized rats	Decreased serum contents of Scr, BUN, TMAO, PCS, and IS; upregulated mRNA and protein expression of occludin and ZO-1		√			√	Wang et al. (2021)
Decoction	*Scutellaria baicalensis* Georgi and *Sophora* japonica L	Spontaneously hypertensive rats	Ameliorated the severity of renal injury induced by hypertension, improved the intestinal barrier function, increased SCFAs, reduced inflammation, decreased IS, and inhibited oxidative stress reactions	√	√	√		√	Guan et al. (2021)
Decoction	Mahuang decoction	Doxorubicin/adenine-induced CKD rats	Mitigated kidney functional and structural impairment; restored the impaired richness and diversity and structure of intestinal microflora	√					Ming et al. (2021)
Decoction	You-Gui pill	Hydrocortisone induced kidney-Yang deficiency syndrome (KYDS) rats	Mediated four kinds of microbes and ten related metabolites; improved the metabolic disorder of KYDS by acting on intestinal microbiota	√					Chen et al. (2019a)
Decoction	Shenqi Yanshen Formula	Adenine-induced CRF rats	Decreased the expression of Scr and BUN; reduced the degree of renal fibrosis; and downregulated the expression of TLR-5, NF-κb, p65, TNF-α, IL-1β, and IL-6	√					Zhang et al. (2022a)
Decoction	Qing-Re-Xiao-Zheng Formula	Diabetic nephropathy rats	Lowered levels of urinary albumin, serum cholesterol, and triglycerides; higher levels of ZO-1 expression and less-damaged colonic mucosa; suppressed the expression of TLR-4 and NF-κB	√				√	Gao et al. (2021b)
Decoction	Rehmanniae Radix Preparata and Corni Fructus	Adenine-induced CKD rats	Elevated the abundance of beneficial bacteria; decreased the opportunistic pathogen; and identified nine putative biomarkers involving in phenylalanine, tyrosine, and tryptophan biosynthesis and tyrosine metabolism	√					Zhang et al. (2021c)
	Rehmanniae Radix Preparata and Corni Fructus	Adenine-induced CRF rats	Reduced the serum contents of Scr, BUN, TMAO, PCS, and IS, upregulated mRNA and protein expressions of occludin and ZO-1 in the ileum tissue		√			√	Zhang et al. (2021d)
Decoction	*Astragalus membranaceus* and *Salvia miltiorrhiza*	Cyclosporin A-induced CRF rats	Alleviated renal fibrosis and metabolism by driving probiotics and regulating butanoate metabolism and tryptophan metabolism	√	√				Han et al. (2020)
Decoction	Bekhogainsam decoction	Streptozotocin-induced diabetic nephropathy rats	Prevented physiological and serological changes, structural damage, and kidney dysfunction; affected the flora composition; acted through PI3K/Akt and MAPK-related protein targets	√					Meng et al. (2020)
Decoction	Tangshen formula	Diabetic nephropathy rats	Inhibited diabetic renal injury, modulated gut microbiota, which decreased levels of LPS and IS, and attenuated renal inflammation	√	√				Zhao et al. (2020)
Decoction	QiDiTangShen granules	Diabetic nephropathy rats	Alleviated renal injuries, altered the gut microbiota composition, and decreased serum levels of TBA and BA profiles	√					Wei et al. (2021)
Decoction	San-Huang-Yi-Shen capsule	Diabetic nephropathy rats	Alleviated the proteinuria, oxidative stress, and inflammatory response in the kidneys; modulated gut microbiota dysbiosis	√					Su et al. (2022)
Decoction + probiotic	*Astragalus mongholicus* Bunge and Panax notoginseng formula + *Bifidobacterium*	5/6 nephrectomized rats	Improved the intestinal flora and protected the intestinal barrier; downregulated macrophage inflammatory response in the kidneys and intestine *via* suppressing Mincle signaling	√			√	√	Rui-Zhi et al. (2020)
Crude extracts	Ethanol and water extract of Danshen	Adenine-induced CRF model	Varied intestinal microbiota	√					Cai et al. (2019) and Cai et al. (2021)
Crude extracts	Total flavones of *Abelmoschus manihot*	5/6 nephrectomized rats	Improved renal injury; remodeled gut microbiota dysbiosis; regulated gut-derived metabolites; inhibited microinflammation; adjusted autophagy-mediated macrophage polarization through AMPK-SIRT1 signaling	√			√		Tu et al. (2020)
Crude extracts	Total phenolic acid from the stems and leaves of *Salvia miltiorrhiza* Bge.	Type 2 diabetic nephropathy mice	Improved the intestinal flora disorder of mice with type 2 diabetic nephropathy; regulated the content of SCFAs in the intestine	√		√			Xu et al. (2021b)
Crude extracts	Total glycoside from the leaves of *Rehmannia glutinosa*	Diabetic nephropathy rats	Restored the dysfunctional intestinal flora to normal; regulated the glycolipid level of db/db mice as well as TGF-β/Smad signaling pathway regulation	√					Dai et al. (2017) and Xu et al. (2020)
Polysaccharides	*Bupleurum* polysaccharides	Diabetic nephropathy rats	Decreased blood Scr, BUN, and blood glucose; modulated the dysbiosis of gut microbiota with higher diversity and gut protective microbiota; improved the gut barrier; and reduced the expression of inflammatory response both in the kidneys and colon	√			√		Feng et al. (2019)
Polysaccharides	*Astragalus* polysaccharides	5/6 nephrectomized rats	Decreased blood Scr, BUN, and 24-h urine protein; repaired the intestinal barrier damage; and regulated the lncRNA Arid2-IR/NF-κB signal axis					√	Yang et al. (2021)
Polysaccharides	Yam polysaccharides	Diabetic nephropathy rats	Decreased body weight, urine protein, Scr, and BUN; regulated the intestinal microecology	√					Zhang et al. (2021a)
Polysaccharides	Moutan Cortex polysaccharide	Diabetic nephropathy rats	Ameliorated hyperglycemia and reduced serum pro-inflammatory mediators, improved intestinal barrier function, and elevated the SCFA contents	√		√		√	Zhang et al. (2022b)
Polysaccharides	*Sporisorium reilianum* polysaccharides	Fructose-induced hyperuricemia mice	Inhibited uric acid biosynthesis and promoted uric acid excretion; downregulated the expression of genes involved in glycolysis/gluconeogenesis metabolic pathways and purine metabolism; decreased the abundances of *Bacteroidetes* and Proteobacteria	√					Wang et al. (2022)
Polysaccharides	*Cordyceps cicadae* polysaccharides	Diabetic nephropathy rats	Improved insulin resistance and glucose tolerance; slowed down the progression of renal interstitial fibrosis; decreased LPS-induced inflammatory cytokine levels and TGF-β1-induced fibroblast activation; and modulated the dysbiosis of gut microbiota	√					Yang et al. (2020a)
Polysaccharides	Polysaccharides from *Armillariella tabescens* mycelia	Type 2 diabetic mice	Ameliorated renal dysfunction; modulated the intestinal microbiota and inflammatory reaction	√				√	Yang et al. (2020b)
Polysaccharides	High-amylose maize resistant starch (HI-MAIZE 260)	5/6 nephrectomized rats	Reduced tubulointerstitial injury and higher population of butyrate-producing bacteria; reduced the abundance of mucin-degrading bacteria	√					Karaduta et al. (2020)
	High-amylose maize resistant starch type 2 (HAM-RS2)	Adenine-induced CKD rats	Host CKD-associated proteins and biomarkers of impaired kidney function were significantly reduced; decreased microbial diversity and an increased ***Bacteroidetes*-to-*Firmicutes*** ratio	√	√				Zybailov et al. (2019) and Kieffer et al. (2016)
	Resistant starch (RS)	Diabetic nephropathy knockout mice	Reshaped gut microbial-ecology; reduced expression of inflammatory cytokines, chemokines, and fibrosis-promoting proteins; identified that GPR43 and GPR109A are critical to SCFA-mediated protection	√		√			Li et al. (2020b)
Polysaccharides	Dietary fermented soy extract and oligo-lactic acid	Adenine-induced CKD rats	Decreased circulating and kidney levels of CKD-associated inflammatory cytokines, circulating levels of kidney injury biomarkers, and kidney levels of stem cell biomarkers; reversed CKD-associated reduction of the cecum *Clostridium leptum* group	√					He et al. (2020)
Polysaccharides	Xylooligosaccharide	Adenine-induced CKD rats	Reversed kidney injuries in CKD mice; decreased alpha diversity; reduced some CKD-enriched bacterial genera; increased cecal SCFA production; and decreased blood PCS	√	√	√			Yang et al. (2018)
Polysaccharides	Sacran	5/6 nephrectomy rats	Increased the number of *Lactobacillus* species, reduced oxidative stress, and serum IS	√	√				Goto et al. (2022)
Polysaccharides	Unmodified and partially hydrolyzed guar gum	Adenine-induced CKD rats	Restored expression of colonic ZO-1, ZO-2, occludin, JAMA, and claudin 7 proteins; higher cecal SCFA and *Lactobacillus* concentrations	√		√		√	Hung and Suzuki (2018)
Polysaccharides	Oligofructose-enriched inulin	Adenine-induced CKD rats	Reduced serum PCS; reduced serum urea and IL-6 levels; and enhanced antioxidant enzyme activity of GPx and SOD in renal tissues of CKD rats		√				Melekoglu et al. (2021)
Synbiotics	GFOB diet (glutamine, dietary fiber, oligosaccharide + *Bifidobacterium longum* strain)	5/6 nephrectomized rats	Lowered Scr, BUN, serum IS, inorganic phosphorus, and intact parathyroid hormone; increased the proportions of *Bifidobacterium* and *Ruminococcus*	√	√				Iwashita et al. (2018)
Natural compound	Emodin	5/6 nephrectomized rats	Altered levels of uremic toxins urea and IS; changed gut microbiota;		√				Zeng et al. (2016)
	Emodin nanoparticle	5/6 nephrectomized rats	improved the kidney function; reduced tubulointerstitial fibrosis; reduced serum IL-1β, IL-6, and LPS levels; improved intestinal barrier functions; downregulated TLR4, MyD88, and NF-κB expression; and regulated microbiota disturbance	√			√	√	Lu et al. (2021b)
	Emodin + deoxycholic acid–chitosan-coated liposomes + *in situ* colonic gel	Unilateral ureteral obstruction (UUO) rats	Attenuated renal fibrosis effectively; restored the gut microbial dysbiosis	√					Xu et al. (2022)
Natural compound	Paramylon	5/6 nephrectomized rats	Attenuated renal function, glomerulosclerosis, tubulointerstitial injury, and podocyte injury; suppressed renal fibrosis, tubulointerstitial inflammatory cell infiltration, and pro-inflammatory cytokine gene expression levels; inhibited the absorption of non-microbiota-derived uremic solutes; and modulated a part of CKD-related gut microbiota	√					Nagayama et al. (2020)
Natural compound	Magnesium lithospermate B	Diabetic nephropathy rats	Decreased 24-h urinary albumin levels and total BAs, CAs, and DCAs and reversed CA:TCA and DCA:CA ratios	√	√				Zhao et al. (2019)
Natural compound	Alisol B 23-acetate	5/6 nephrectomized and unilateral ureteral obstructed rats	Re-established dysbiosis of the gut microbiome, lowered blood pressure, reduced Scr and proteinuria, suppressed expression of RAS constituents, and inhibited the epithelial-to-mesenchymal transition and Smad7-mediated inhibition of Smad3 phosphorylation	√					Chen et al. (2020)
Natural compound	Isoquercitrin	Adenine-induced CKD rats	Inhibited the transport of tryptophan and further reduced gut microbial indole biosynthesis		√				Wang et al. (2020b)
Natural compound	Curcumin	Uric acid nephropathy rats	Attenuated renal pathological lesions and metabolic endotoxemia; increased the relative abundance of bacteria-producing SCFAs; and improved tightly linked protein expression	√	√	√		√	Xu et al. (2021a)
	Docosahexaenoic acid-acylated curcumin diester	Cisplatin-induced acute kidney injury mice	Changed the relative abundance of microbiota related to LPS and TMAO/TMA metabolism; prevented the LPS and TMAO-mediated PI3K/Akt/NF-κB signaling pathway	√	√				Shi et al. (2022)
Natural compound	Resveratrol	Diabetic db/db and db/m mice	Decreased levels of Scr, BUN, and UAER; improved intestinal barrier function and ameliorated intestinal permeability and inflammation; decreased the ***Firmicutes*-to-*Bacteroidetes*** (F/B) ratio	√				√	Cai et al. (2020)

**TABLE 2:** Clinical trial of natural products from plants and microorganisms in the treatment of CKD patients by modulating gut microbiota.

Intervention type	Intervention	Country	Study type	Population	Result	Group size		Study duration	Reference
						CKD	Con		
Prebiotic	ß-Glucan prebiotic	South Africa	Single-center, single-blinded study	CKD patients with stage 3–5	Altered total uremic toxin and free pCG levels; lowered beta diversity of gut microbiota	30	29	14 weeks	Ebrahim et al. (2022)
Prebiotic	Fructo-oligosaccharide	Brazil	Double-blind, placebo-controlled, randomized trial	Non-diabetic, non-dialysis-dependent CKD patients	Decreased serum total ΔPCS and serum-free Δ%PCS	24	26	3 months	Ramos et al. (2019)
Prebiotic	p-Inulin (oligofructose-enriched inulin)	United States of America	Multicenter, non-randomized, crossover feasibility study	CKD patients on hemodialysis	Increased intraparticipant microbiome diversity during and after treatment	13	0	28 weeks	Raj et al. (2021)
	Oligofructose-enriched inulin	Belgium	Single-center, non-randomized, open-label study	CKD patients on hemodialysis	Reduced p-cresyl sulfate generation rates and serum concentrations	22	0	4 weeks	Meijers et al. (2010)
Prebiotic	Inulin-type fructan	China	Randomized, double-blind, placebo-controlled, crossover trial	Patients with peritoneal dialysis	Restricted the increase in gut microbiome-generated indole	8	7	36 weeks	Li et al. (2020a)
Prebiotic	Resistant starch	Brazil	Randomized, double-blind, placebo-controlled clinical trial	CKD patients on hemodialysis	Reduced inflammatory factors RANTES, PDGF, and CXCL10	8	8	4 weeks	De Paiva et al. (2020)
	Resistant starch cookies	Brazil	Randomized, double-blind, placebo-controlled trial	CKD patients on hemodialysis	Reduced IL-6, TBARS, and IS plasma levels; increased fiber intake	19	19	4 weeks	Esgalhado et al. (2018)
	Resistant starch type 2 cookies	Brazil	Randomized, double-blind, placebo-controlled trial study	Hemodialysis patients	Altered SCFA producers in the gut microbiota	10	10	4 weeks	Kemp et al. (2021)
	Resistant starch supplementation	Brazil	Longitudinal, randomized, double-blind, placebo-controlled clinical trial	Hemodialysis patients	No effects on plasma indole 3-acetic acid and aryl hydrocarbon receptor mRNA expression	22	20	4 weeks	Azevedo et al. (2020)
	Amylose resistant starch (HAM-RS2)	Iran	Double-blind, parallel, randomized, placebo-controlled trial	End-stage renal disease patients	Elevated *Faecalibacterium* and decreased systemic inflammation	9	11	2 months	Laffin et al. (2019)
	High-amylose resistant starch (HAM-RS2)	Iran	Double-blind, randomized, parallel, placebo-controlled trial	Hemodialysis patients	Reduced levels of inflammatory and oxidative markers	22	22	2 months	Tayebi Khosroshahi et al. (2018)
	Unripe banana flour (UBF—48% resistant starch)	Brazil	Randomized, double-blind, placebo-controlled, crossover trial	Patients undergoing automated peritoneal dialysis	Decrease in IS was only found in the subgroup of participants taking 21 g/day of UBF.	21	22	12 weeks	De Andrade et al. (2021)
Prebiotic	Low-protein diet and inulin	Italy	Longitudinal, prospective, controlled, interventional study	Patients with stage 3–4 CKD	Modified gut microbiota and modulated inflammatory and metabolic parameters	9	7	6 months	Lai et al. (2019)
Bioactive compound	Curcumin supplementation	Brazil	Longitudinal, randomized, double-blind, controlled trial	Patients with CKD on hemodialysis	Reduced p-CS plasma levels	14	14	3 months	Salarolli et al. (2021)
	Curcumin supplementation (Meriva^®^)	Italy	Clinic pilot study	CKD from stage 3a–4	Reduced plasma CCL-2, IFN-γ, IL-4, and lipid peroxidation; lowered *Escherichia* and *Shigella*, while higher *Lachnoclostridium*	24	20	6 months	Pivari et al. (2022)
Bioactive compound	Trans-resveratrol supplementation	Brazil	Randomized, double-blind, placebo-controlled, crossover trial	Non-dialyzed patients with stages 3 and 4 CKD	Did not reduce the plasma levels of IS, pCS, and IAA	10	10	16 weeks	Alvarenga et al. (2022)
Bioactive compound	Cranberry dry extract supplementation	Brazil	Randomized, double-blind, placebo-controlled study	Non-dialysis patients with stages 3 and 4 CKD	Did not reduce the LPS and uremic toxin plasma levels	15	15	2 months	Teixeira et al. (2022)
Herbal medicine	Jian Pi Qu Shi Formula	Beijing, China	Single-center, randomized, controlled trials	Patients with idiopathic membranous nephropathy (IMN)	Declined 24-h urinary protein; regulated intestinal flora in patients with IMN	15	10	6 months	Lang et al. (2020)
Herbal medicine	Shenqi Dihuang Decoction	Sichuan, China	Clinical observation study	Patients with diabetic kidney disease	Reduced proteinuria, protected renal function, restored the balance of intestinal flora, and alleviated chronic inflammatory	75	72	3 months	Du et al. (2021b)
Herbal medicine	Shen-Shuai-Ning (SSN)	Shanghai, China	Randomized, single-blinded study	Uremic patients undergoing peritoneal dialysis	Decreased serum total IS level effectively	30	30	12 weeks	Chen et al. (2018)
Herbal medicine	Fushen Granule	Tianjing, China	Randomized, controlled clinical trial	Patients with peritoneal dialysis-related peritonitis	Enriched beneficial bacteria associated with metabolism	12	14	6 months	Lin et al. (2021b)
Herbal medicine	Zicuiyin decoction	Tianjing, China	Multicenter, parallel-control, open-label, randomized clinical trial	Type 2 diabetes mellitus and DKD	Declined eGFR and gut microbiota dysbiosis	44	44	2 months	Liu et al. (2022)
Synbiotic	High-resistant starch fiber supplement + the probiotic component	Australia	Double-blind, placebo-controlled, randomized, controlled trial	Adults with stage 3–4 chronic kidney disease	Altered the stool microbiome with an enrichment of *Bifidobacterium* and *Blautia* spp.; reduced eGFR; and increased Scr concentration	35	33	12 months	McFarlane et al. (2021)
Synbiotic	Symbiotic gel (Nutrihealth^®^)	Mexico	Randomized, double-blind, placebo-controlled clinical trial	Patients with end-stage renal disease	Increased *Bifidobacterium* counts	8	10	2 months	Cruz-Mora et al. (2014)
Synbiotic	Synbiotic Probinul-Neutro^®^	Italy	Double-blind, randomized placebo study	Non-dialyzed CKD patients on 3–4 stages	Lowered total plasma p-cresol concentrations	18	12	1 month	Guida et al. (2014)
Synbiotic	Synbiotic NatuREN G (Farmalabor^®^ SRL)	Italy	Randomized, single-blind, placebo-controlled pilot trial	Stage 3b–4 CKD patients	Decreased free IS; reduced small intestinal permeability; and ameliorated abdominal pain and constipation symptoms	23	27	2 months	Cosola et al. (2021)
Synbiotic	Synbiotic capsules (GeriLact brand; Zist Takhmir Company, Tehran, Iran)	Iran	Randomized, double-blind, placebo-controlled clinical trial	Patients on hemodialysis	Increased indoxyl sulfate and parathyroid hormone levels	21	21	2 months	Mirzaeian et al. (2020)
Synbiotic	Combination of inulin, fructo-oligosaccharides, and galacto-oligosaccharides and the probiotic component	Australia	Randomized, double-blind, placebo-controlled crossover trial	Non-dialyzed patients with stage 4 or 5 CKD	Altered the stool microbiome with enrichment of *Bifidobacterium* and depletion of Ruminococcaceae; increased albuminuria; and reduced serum PCS	17	20	16 weeks	Rossi et al. (2016)

## 2 Animal studies of natural products for the treatment of CKD associated with gut microbiota

### 2.1 Remodulation of gut microbiota structure

**FIGURE 1:**
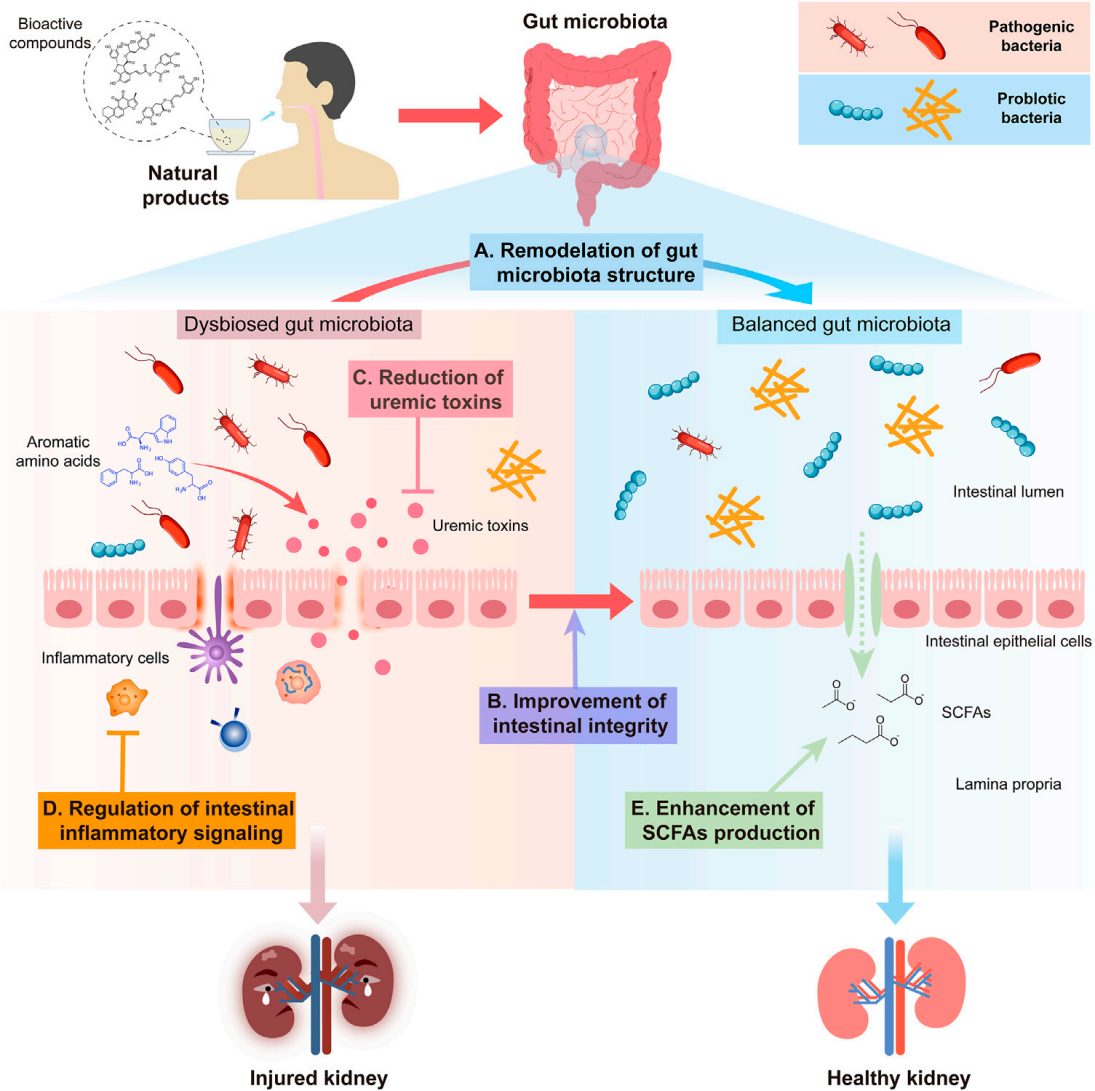
Schematic graphic representation of strategies of gut microbiota as an alternative target for natural products from plants and microorganisms in the treatment of CKD. The strategies of these anti-CKD effects of herbal medicine include **(A)** remodulation of the gut microbiota structure; **(B)** improvement in intestinal integrity; **(C)** reduction of uremic toxins; **(D)** regulation of intestinal inflammatory signaling; and **(E)** enhancement of SCFA production.

Natural products from plants and microorganisms exhibited their prebiotic-like effects by remodulating the gut flora structure (Figure 1A).

#### 2.1.1 Firmicutes and Bacteroidetes ratio

At the phylum level, ***Firmicutes* and *Bacteroidetes*** are the representative intestinal microbiota. For diabetic nephropathy, the ***Firmicutes*-to-*Bacteroidetes*** ratio (F/B ratio) has an important influence on obesity (Etxeberria et al., 2015), body mass index (Koliada et al., 2017), and glucose levels (Remely et al., 2016). Some studies found that the F/B ratio was significantly increased in the diabetic nephropathy model. Phytochemical resveratrol treatment significantly reduced body and kidney weights but not the fasting blood sugar (FBS) of db/db mice, which were associated with the decrease in the F/B ratio after resveratrol treatment (Cai et al., 2020). Qing-Re-Xiao-Zheng formula (Gao Y. et al., 2021), *Cordyceps cicadae* polysaccharides (Yang J. et al., 2020), and San-Huang-Yi-Shen capsule (Su et al., 2022) significantly lowered serum glucose and the ratio of ***Firmicutes*-to-*Bacteroidetes*** in mice with diabetic kidney disease. While treatment with QiDiTangShen granules and Tangshen formula had no significant effects on the F/B ratio in db/db mice, this maybe partly explains why they do not alter body weight and glycemic levels in those mice (Zhao et al., 2020; Wei et al., 2021). For other nephropathy models, the value of F/B was decreased after the intervention of *Astragalus membranaceus* and *Salvia miltiorrhiza* in CsA-induced chronic nephrotoxicity (Han et al., 2020). The combination of *Scutellaria baicalensis* Georgi (SB) and *Sophora japonica* L. (SL) lowered the F/B ratio in spontaneously hypertensive rats (Guan et al., 2021). Resistant starch HAM-RS2 (HI-MAIZE 260) resulted in a decrease in the F/B ratio in the adenine-induced CKD model (Kieffer et al., 2016; Zybailov et al., 2019; Li Y. J. et al., 2020).

However, the definite association between F/B ratio and biochemical indicators of diabetes is not well established and still needs to be further studied (Mokhtari et al., 2021). On the contrary, the ***Firmicutes*-to-*Bacteroidetes*** ratio was found to have declined significantly in some studies of diabetic nephropathy (Dai et al., 2017; Zhao et al., 2019; Chen et al., 2020). The Shenyan Kangfu tablet (Chen et al., 2020) and *Rehmannia glutinosa* leaves total glycoside (Dai et al., 2017; Xu et al., 2020) reduced stimulated blood glucose and increased the F/B ratio in db/db mice. Magnesium lithospermate B did not affect FBG levels and increased the F/B ratio slightly in the streptozotocin (STZ)-induced mice (Zhao et al., 2019). In the other CKD models, the decreased F/B ratio was also observed in adenine-induced CKD rats, and the combination of Rehmanniae Radix Preparata (RR) and Corni Fructus (CF) significantly altered the ratio trend (Zhang et al., 2021c & 2021d). After supplementation with dietary docosahexaenoic acid-acylated curcumin diester and curcumin, the ratio of F/B was remarkably elevated in acute kidney injury mice (Shi et al., 2022). *Sporisorium reilianum* polysaccharides could notably increase the ratio of F/B in hyperuricemic mice (Wang et al., 2022).

#### 2.1.2 Deleterious bacteria

The phylum ***Proteobacteria*** was mainly composed of opportunistic pathogens, which were found at low levels (less than 4%) in the normal group. The expansion of ***Proteobacteria*** abundance is considered a hallmark for dysbiosis (Shin et al., 2015). For CKD rats, the abundance of Enterobacteriaceae was largely increased (Chen et al., 2020; Xu X. et al., 2021; Ji et al., 2021; Ming et al., 2021), which is responsible for the production of uremic toxins, such as indoxyl sulfate (IS) and p-cresol sulfate (PCS) (Li and Young, 2013). Emodin *via* colonic irrigation (Zeng et al., 2016) and *Astragalus* polysaccharide (Yang et al., 2021) remarkably decreased *Escherichia coli*, which is positively correlated with both urea and IS. The relative abundance of *Escherichia* and *Shigella* from family Enterobacteriaceae was significantly increased in the injured kidney mice which were restored by the intervention of curcumin (Xu X. et al., 2021) and alisol B 23-acetate (Chen et al., 2020). Tangshen formula-treated rats exhibited decreases in the Enterobacteriaceae family (OTU167 and OTU218) in DN rats (Zhao et al., 2020). As a pathogenic genus from phyla Proteobacteria, *Desulfovibrio* was proved to reduce sulfate to intestinal toxin hydrogen sulfide (Kushkevych et al., 2019). The increased number of *Desulfovibrio* in the CKD rats was strongly decreased by You-Gui pill (Chen R. et al., 2019) and the combination of RR and CF treatment (Zhang et al., 2021c; Zhang et al., 2021d).

The phylum ***Actinobacteria*** is the most important flora that causes intestinal inflammation and stimulates multiple inflammatory reactions (Burge et al., 2019). Oral administration of Bekhogainsam decoction resulted in a significant decrease in ***Actinobacteria*** in the STZ-induced DN mice (Meng et al., 2020). The abundance of ***Actinobacteria*** was increased in CKD rats, and the prebiotic acacia gum (Lakshmanan et al., 2021) and Jian-Pi-Yi-Shen decoction (Zheng et al., 2020) treatment successfully reversed those levels.

#### 2.1.3 Beneficial bacteria

The relative abundance of bacteria producing SCFAs, including *Lactobacillus*, *Akkermansia*, *Lachnospiraceae*, and *Ruminococcaceae*, was significantly lower in CKD animal models. *Lactobacillus*, as a lactic acid-producing probiotic, has been observed to decrease in CKD patients and different CKD models (Zeng et al., 2016; Nagayama et al., 2020). Its increase could protect kidney function by reducing the accumulation of urinary toxins such as IS, repairing the intestinal barrier, and improving inflammation and oxidative stress (Zhu et al., 2021; Kim et al., 2022; Tungsanga et al., 2022). Treatment with adenine reduced the Lactobacillaceae count, whereas additional supplementation with acacia gum (Al-Asmakh et al., 2020) and guar gum dietary fibers (Hung and Suzuki, 2018) reversed this effect in the intestinal tract. Compared to spontaneously hypertensive rats, the relative abundance of *Lactobacillus* was increased in the group of SB and SL treatments (Guan et al., 2021). *Lactobacillus* were relatively less abundant in STZ-induced diabetic nephrotoxicity (Su et al., 2022), which was increased by high-dose San-Huang-Yi-Shen capsule. Emodin *via* colonic irrigation (Zeng et al., 2016), sacran (Goto et al., 2022), and alisol B 23-acetate (Chen et al., 2020) remarkably increased *Lactobacillus* in 5/6 nephrectomized (Nx) mice. *A. membranaceus* and *S. miltiorrhiza* (Han et al., 2020), Moutan Cortex polysaccharide (Zhang M. et al., 2022), polysaccharides from *Armillariella tabescens* mycelia (Yang R. et al., 2020), and *Cordyceps cicadae* polysaccharides (Yang J. et al., 2020) not only recalled the content of *Lactobacillus* but also significantly increased the abundance of *Akkermansia* in chronic nephrotoxicity models. *Akkermansia*, a genus of the phylum ***Verrucomicrobia***, is also known as a beneficial gut microbe because of its advantage in the maintenance of gut integrity (Taherali et al., 2018; Cani et al., 2022). *A. muciniphila* slows down the development and progression of diabetes, obesity, and IBD in mice (Rodrigues et al., 2022), which was enriched by the total flavones of *Abelmoschus manihot* treatment in CRF rat models (Tu et al., 2020).

A number of studies have illustrated that Ruminococcaceae and Lachnospiraceae families have been discovered to be related to the enhancement of immunological response (Davar et al., 2021), the improvement in renal function (Xu et al., 2017; Xu X. et al., 2021), and primary bile acid production (Zhao et al., 2019). Furthermore, the amount of Ruminococcaceae, which contains many butyrate-producing genera, such as *Ruminococcaceae* UCG-014 and *Ruminococcus* 1, was greatly enhanced by RR and CF (Zhang et al., 2021c; Zhang et al., 2021d), *Bupleurum* polysaccharides (Feng et al., 2019), and curcumin (Xu X. et al., 2021) in CKD rats. Resistant starch slowed the progression of renal injury by enriching the butyrate producers *Ruminococcus torque*s and *Eubacterium ruminantium* in 5/6 nephrectomy CKD rats (Karaduta et al., 2020). The Lachnospiraceae family, which was reduced in the CKD models, was increased by emodin nanoparticle treatment (Lu Z. et al., 2021) and yam polysaccharide (Zhang W. et al., 2021). The acetic acid-producing genera *Lachnospiraceae* UCG-001 and the *Lachnospiraceae* NK4A136 group, which significantly decreased in CKD rats, were drastically enriched following the intervention of RR and CF (Zhang et al., 2021c and 2021d) and dietary docosahexaenoic acid-acylated curcumin diester or curcumin (Shi et al., 2022). The genus *Lactonifactor* was significantly increased in the total flavones of *Abelmoschus manihot* treatment of the potassium oxonate-induced CRF rat model (Tu et al., 2020).

For other probiotic microbes, Mahuang decoction (Ming et al., 2021) and resistant starch diet (Li Y. J. et al., 2020) could promote the expansion of SCFA-producing bacteria of the genus *Prevotella*, which are disrupted in CKD models. *Prevotella*-9 were increased by the SB and SL in spontaneously hypertensive rats. Butyric acid-producing bacteria Bacteroidales S24-7 were enriched with the administration of Mahuang decoction (Ming et al., 2021), You-Gui pill (Chen R. et al., 2019), and rhubarb enema (Ji et al., 2020) in CKD rats. *Coprococcus* play an important role in body health through its production of acetate acid and vitamin B (Nogal et al., 2021) and have become significantly more abundant in the group treated with paramylon (Nagayama et al., 2020) and Jian-Pi-Yi-Shen decoction (Zheng et al., 2020) than the 5/6 Nx group. Tangshen formula (Zhao et al., 2020), rhubarb enema treatment (Ji et al., 2021), fermented soybean product (Koji polysaccharides ^®^) (He et al., 2020), *Astragalus* polysaccharide (Yang et al., 2021), Coptidis Rhizoma extracts (Cui et al., 2018), and a resistant starch diet (Li Y. J. et al., 2020) enhanced the *Bifidobacteriaceae* genus that was decreased in CKD models. The proportions of *Bifidobacterium* and *Ruminococcus* were significantly elevated in the GFOB diet-fed 5/6 Nx rats (Iwashita et al., 2018). The abundance of *Lachnoclostridium* was significantly raised in the *Sporisorium reilianum* polysaccharide-treated hyperuricemia mice (Wang et al., 2022). Shenqi Yanshen Formula significantly modified the dysbiosis of gut microbiota by increasing the abundance of Succinivibrionaceae in the adenine-induced CKD model (Zhang L. et al., 2022).

However, some studies showed that the abundance of SCFA-producing bacteria was enhanced in the kidney injury model, which is contrary to the aforementioned examples. It may be due to the different strains even within the same family having existential discrepancies in their responses to one kind of nature products, which cannot be distinguished by 16 S rDNA sequencing. The abundance of *Lactobacillus* and *Bacteroides* enriched in the diabetic mice, which were significantly reduced by QiDiTangShen granules (Wei et al., 2021) and the Shenyan Kangfu tablet (Chen et al., 2021). The IgAN rat group had higher abundance of Lachnospiraceae, Lactobacillaceae, and Bacteroidaceae than the normal group, which was reduced upon Zhen Wu Tang intervention (Li et al., 2021). Following treatment with total flavones of *Abelmoschus manihot*, the abundance of *Bacteroidales* and *Lactobacillales* was decreased, which were enriched in potassium oxonate-induced CRF rat models (Tu et al., 2020). The relative abundance of *Bacteroidetes* and *Bacteroidales* S24-7 in db/db mice was significantly increased, which was significantly reduced by *Rehmannia glutinosa* leaves total glycoside (Dai et al., 2017; Xu et al., 2020). The *Ruminococcus* genus (OTU82, OTU230, and OTU51) that increased in the 5/6 Nx group was found to be associated with kidney injury (Zeng et al., 2016; Zhao et al., 2020). The water extract of Dansen (Cai et al., 2019; Cai et al., 2021) and alisol B 23-acetate (Chen et al., 2020) could downregulate the *Ruminococcus* induced by adenine-induced injury. Gum acacia treatment reduced the genus *Akkermansia*, which was found enriched in the adenine-induced CKD rats (Lakshmanan et al., 2021) and the 5/6 Nx group (Ji et al., 2020).

In a word, natural products from plants and microorganisms could stimulate the growth of beneficial bacteria and inhibit the colonization of potential or opportunistic pathogens in the intestine.

### 2.2 Improvement in intestinal integrity

The intestinal epithelial barrier is formed by epithelial cells to prevent enteral substances from entering the rest of the body, and it plays a crucial role in resisting the colonization of exogenous microorganisms. Aberrant physical conditions induced by the gastrointestinal dysbiosis of CKD lead to disruption of the intestinal muco/sa (Figure 1B). Damaged intestinal barriers increase intestinal permeability, which permits the pathogenic bacteria or endotoxin (such as lipopolysaccharide (LPS)) to translocate across the intestinal epithelial cells into the circulation of the blood and lymphoid system (Boyapati et al., 2016). Therefore, maintaining the integrity and function of the intestinal epithelial barrier has therapeutic significance for CKD.

Tight junction proteins, such as zonula occludens-1 (ZO-1), claudin-1, and occludin, play an indispensable role in maintaining the permeability of the intestinal epithelial barrier. Once the expression of tight junction proteins in intestinal mucosa decreases, the paracellular permeability would increase and the tight junctions would be destroyed, resulting in intestinal barrier damage (Paone and Cani, 2020). Decreased expression of tight junction proteins was detected in different nephropathy models. Couplet medicine of *Rheum palmatum*–*S. miltiorrhiza* can effectively improve intestinal barrier function in CRF rats by upregulating the expression of occludin and ZO-1 in the ileum tissue of the 5/6 nephrectomy model (Wang et al., 2021). Higher levels of ZO-1 expression and less-damaged colonic mucosa with a lower serum level of FITC-dextra, a marker of intestinal permeability, implied the beneficial role of Qing-Re-Xiao-Zheng formula for gut barrier integrity in the diabetic nephrectomy model (Gao Y. et al., 2021). Fermentable dairy fiber unmodified guar gum (GG) restored colonic barrier integrity by higher expression of colonic ZO-1, occludin, and claudin-1 proteins in adenine-induced CKD mice (Hung and Suzuki, 2018). *Astragalus* polysaccharide (Yang et al., 2021), rhubarb enema decoction (Ji et al., 2020), the combination of *Astragalus mongholicus* Bunge and Panax notoginseng formula with *Bifidobacterium* (Rui-Zhi T, et al., 2020) and alisol B 23-acetate (Chen et al., 2020) treatment restored intestinal epithelial tight junctions and reduced intestinal permeability with upregulated expression of ZO-1, occludin, and claudin-1 proteins in 5/6 Nx rat models. Polysaccharides from *Armillariella tabescens* mycelia (Yang R. et al., 2020), *Bupleurum* (Feng et al., 2019; Liu et al., 2019), and Moutan Cortex (Zhang M. et al., 2022) improved the gut barrier by increasing the protein expression of occludin and claudin-1 in the colonic tissues of the diabetic nephropathy rats. Resveratrol (Cai et al., 2020) and curcumin supplementation (Xu X. et al., 2021) and the combination of SB and SL (Guan et al., 2021) protected the intestine epithelial barrier by substantially recovering ZO-1 and claudin-1 protein expression in CKD models. *A. membranaceus* and *S. miltiorrhiza* recovered intestinal permeability by enhancing the expression of ZO-1 in CsA-induced chronic nephrotoxicity (Han et al., 2020). Thus, improving the intestinal barrier with natural products from plants and microorganisms can effectively alleviate intestinal inflammation and renal fibrosis.

### 2.3 Reduction of uremic toxins

Intake of choline, tyrosine, and tryptophan increases the amounts of trimethylamine (TMA), IS, hydrogen sulfide, and indole produced by intestinal bacteria. After absorption, these compounds are further metabolized in the liver to generate the typical uremic toxin trimethylamine N-oxide (TMAO), PCS, and p-indoxyl sulfate. These toxins, which cannot be removed efficiently even by hemodialysis and would accumulate in advanced CKD patients (Wu et al., 2020), are highly related to the progression and mortality of multiple cardiovascular diseases (CVDs) and CKDs (Lin et al., 2015; Cheng et al., 2020; Zhang Y. et al., 2021; Lim et al., 2021) (Figure 1C).

Obvious studies showed that natural products could reduce the production of uremic toxin. Orally administered Tangshen formula significantly inhibited diabetic renal injury by decreasing the amount of bacteria producing the precursor of IS and then the serum levels of LPS and IS (Zhao et al., 2020). Rhubarb enema granule treatment ameliorated tubulointerstitial fibrosis in the kidneys of CKD rats, most likely by alleviating circulating TMAO and IS levels (Lu et al., 2015; Ji et al., 2021). The small molecule compound piceatannol (PIC) could inhibit the synthesis of uremic toxin precursors in *Bacillus*, thereby reducing the accumulation of IS and PCS in CKD mice (Li et al., 2022). The DHA-acylated curcumin diester treatment remarkably lowered the LPS and TMAO/TMA of AKI mice by decreasing the relative abundance of intestinal microflora with their metabolism (Shi et al., 2022). Emodin, an abundant anthraquinone in the roots and bark of the traditional Chinese medicine rhubarb (Da Huang), has been demonstrated to reduce uremic toxins and is used in China for the treatment of CKD (Li et al., 2015; Sun et al., 2019). Emodin *via* colonic irrigation (ECI) remodeled gut microflora and decreased the levels of urea and IS in CKD rats (Zeng et al., 2016). Deoxycholic acid–chitosan-coated liposomes could enhance the renoprotective effect of emodin (Xu et al., 2022).

Supplementation with amylose resistant starch HAM-RS2 (Kieffer et al., 2016) could reduce urine IS and p-cresol (PC) in adenine-induced CKD rats. Sulfated polysaccharide sacran (Goto et al., 2022) and GFOB diet (containing prebiotics such as glutamine, dietary fiber, and oligosaccharide and probiotic strain *Bifidobacterium longum*) (Iwashita et al., 2018) completely diminished serum levels of IS in 5/6 Nx rats, whereas xylooligosaccharide supplementation could decrease serum levels of IS and PCS by altering microbial tyrosine metabolism (Yang et al., 2018). The combination of SB and SL attenuated higher serum levels of IS and severe oxidative stress in the kidneys of spontaneously hypertensive rats (Guan et al., 2021). Oligofructose-enriched inulin significantly reduced serum PCS and urea and enhanced antioxidant enzyme activity in renal tissues of CKD rats (Melekoglu et al., 2021). Further studies revealed that transporting tryptophan during indole production may be an important inhibition target for natural products. Huangkui capsule (water extract of *Abelmoschus moschatus*) inhibited the tryptophan transport in the main indole-synthesizing bacteria ***Enterobacteriaceae***, resulting in the decease of uremic toxin IS production in CKD rats (Wang et al., 2019). Huangkui Siwu Formula (HKSWF), containing *Abelmoschus moschatus*, *Astragalus mongholicus*, *Polygonum cuspidatum*, and *Curcuma longa* L., could inhibit the conversion of p-cresol into urotoxin PCS in the liver and directly inhibit the oxidative pathway of tyrosine and decrease the PC production in CKD rats (Lu et al., 2020; Lu J. et al., 2021). As a natural flavonoid isolated from *Bidens bipinnata* L., isoquercitrin (quercetin-3-O-d-glucopyranoside) could disturb microbiota-mediated indole production by inhibiting the transport of exogenous tryptophan into indole-synthesizing bacteria and further reducing indole biosynthesis (Wang Y. et al., 2020). To sum up, modulation of uremic toxin production by gut microbiota is one of the main strategies of the mechanisms of natural products from plants and microorganisms to delay CKD progression.

### 2.4 Regulation of intestinal inflammatory signaling

The dysbiosis of gut microbiota induced by kidney injury can affect the intestinal microenvironment through modulation of the inflammatory process in the gastrointestinal tract (Chi et al., 2021) (Figure 1D). For example, the abundance of Proteobacteria and the Gram-negative family, including pathogenic ***Enterobacteriaceae***, ***Vibrionaceae***, and ***Pseudomonadaceae***, was found to increase significantly in the CKD patients (Jiang et al., 2017). They can secrete pro-inflammatory elements, such as the endotoxin LPS, which would stimulate both local intestinal and systematic chronic inflammation when accumulating in the blood (Ciesielska et al., 2021). These trigger TLR-4 signaling activation and the subsequent release of inflammatory cytokines (Wang et al., 2014). A&P combined with *Bifidobacterium* could protect nephridial tissue against CKD by downregulating Mincle/NF-κB inflammatory signaling transduction in the intestine (Rui-Zhi et al., 2020). Emodin-NP *via* colonic irrigation (Lu Z. et al., 2021) and rhubarb enema treatment (Ji et al., 2020) remarkably alleviated microbiota disturbance in CKD rats and inhibited the expression of TLR-4, MyD88, and NF-κB of the TLR-4 signaling pathway in the intestinal tract of the 5/6 Nx model. *Bupleurum* polysaccharides could significantly reduce the expression of HMGB1/TLR-4/NF-κB/IL-6 inflammatory factors in the colon tissue (Feng et al., 2019). In addition, resveratrol treatment significantly decreased IFN-γ and TNF-α levels in the intestine of db/db mice (Cai et al., 2020). Treatment with polysaccharides from *Armillariella tabescens* mycelia decreased the concentrations of colonic pro-inflammatory cytokines TNF-α and IL-1β (Yang R. et al., 2020). Therefore, reducing the intestinal inflammatory reaction with natural products from plants and microorganisms could contribute to attenuating the systematic chronic inflammation in CKD.

### 2.5 Enhancement of SCFA production

SCFAs (acetate, propionate, butyrate, etc.) are generated from the fermentation of various types of cellulose by SCFA-producing gut microbiota such as *Prevotella*, *Faecalibacterium*, *Bacteroides*, and *Akkermansia* under anaerobic conditions (Koh et al., 2016) (Figure 1E). The easily absorbed SCFAs exert beneficial physiological effects on the host *via* epigenetic modification or the G protein-coupled receptors, including providing energy for intestinal epithelial cells, promoting their proliferation, maintaining intestinal barrier function, maintaining intestinal homeostasis, and improving immune tolerance (Koh et al., 2016; Parada Venegas et al., 2019). For example, gut γδ T cells play indispensable roles in host defense and regulation of intestinal chronic inflammation. Propionate could inhibit γδ T cells producing interleukin-17A (IL-17) in a histone deacetylase-dependent manner (Dupraz et al., 2021). Compared to the non-CKD controls, SCFAs were significantly decreased in patients with severe CKD (Wu et al., 2020).

Dietary administration of gum acacia water extracts in CKD rats improved their renal function by modulating the microbiome composition and plasma levels of ethanoic acid, propionic acid, butanoic acid, and pentanoic acid (Al-Asmakh et al., 2020; Lakshmanan et al., 2021). It has been demonstrated that the combination of SB and SJ is effective in improving kidney injury caused by hypertensive in clinic. The combination of SB and SJ treatments increased SCFA production, upregulated the expression of receptor GPR41, and downregulated the expression of Olfr78 in male spontaneously hypertensive rats (Guan et al., 2021). *A. membranaceus* and *S. miltiorrhiza* increased serum SCFA content by enhancing the growth of butyric acid- and lactic acid-producing probiotics, especially *Lactobacillus* and *Akkermansia* (Han et al., 2020). Total phenolic acid from the stems and leaves of *S. miltiorrhiza* could ameliorate the intestinal microflora disorder of mice with diabetic nephropathy and regulate the content of SCFAs *via* adjusting the amount of some SCFA-producing bacteria in the intestine (Xu Z. et al., 2021). Furthermore, resistant starch (Li Y. J. et al., 2020), guar gum (Hung and Suzuki, 2018), xylooligosaccharide (Yang et al., 2018), and Moutan Cortex polysaccharide (Zhang M. et al., 2022) could also increase cecal SCFA production. Altering SCFAs with natural products from plants and microorganisms has proved to be another potential therapeutic strategy to mitigate kidney injury and slow the progression of renal decline.

**FIGURE 2:**
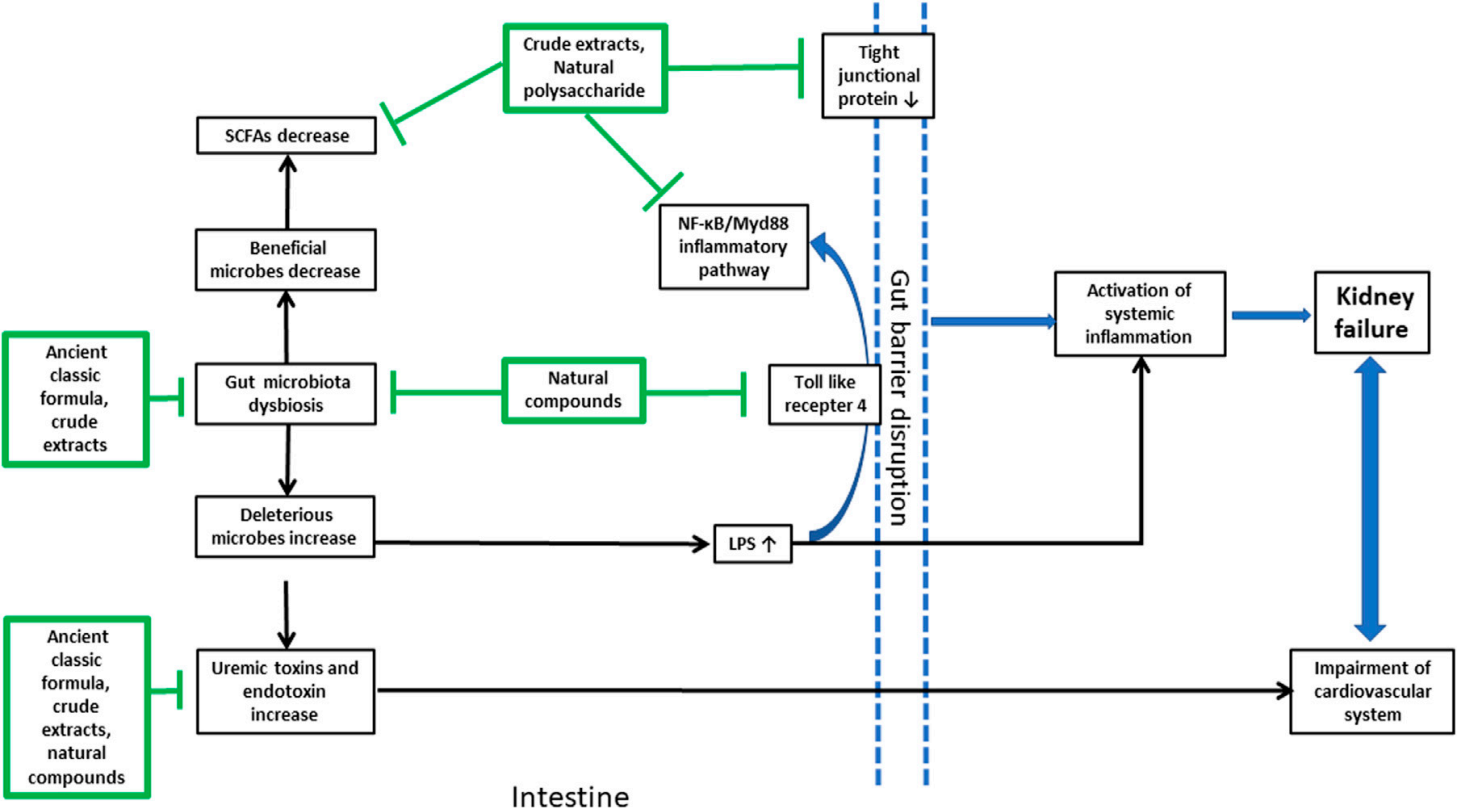
Brief summary of the natural products from plants and microorganisms used in the treatment of CKD and their multi-targets in the gut microenvironment. The arrow line with a pointed tail means promoting effects and that with a flat tail means inhibitory effects.

In summary, natural products from plants and microorganisms were effective in the treatment of different kinds of CKD by modulating the gut microbiota in a multi-channel and multi-target way (Figure 2). Some ancient classic formula, crude extracts from herbal medicine, and dietary fiber could change the imbalance within different intestinal floras of CKD by increasing the number of probiotics and reducing the amount of deleterious microbes. Natural polysaccharides, which led to an increase in SCFAs, decreased the intestinal mucosal barrier permeability by enhancing the tight junction expression of the intestinal epithelium. Also, these prevented pathogenic bacterial growth and large amount of endotoxin LPS produced by Gram-negative bacilli over-proliferated in the intestine, translocating into the blood to enhance systematic inflammatory responses. Some crude extracts and natural polyphenol compounds could disrupt immune responses by inhibiting the inflammatory signal transduction produced by pathogenic bacteria-stimulating dendritic cells in the colon; some herbal medicines, flavonoid and anthrone compounds, and polysaccharides from plants and microorganisms could inhibit the production of IS, PCS, and TMAO fermented by intestinal pathogenic bacteria. Moreover, the restoration of dysbiosis of gut microbiota was not independent from attenuating metabolic endotoxins and inhibiting the inflammatory signaling pathway.

## 3 Clinical trial

Besides animal experiment, accumulating clinical trials have shown that natural products from plant and microorganism have an effect on the reduction of proteinuria and improvement of renal function by modifying the gut microbiota (Table 2). The first benefit of administering natural products during CKD is recovery of the dysbiosis of gut microflora. The Jian Pi Qu Shi Formula, made of *Astragalus membranaceus* Bge., *Codonopsis pilosula*, and other eight herbal medicines, was shown to regulate intestinal flora in the pilot trial of patients with idiopathic membranous nephropathy (Lang et al., 2020). A cohort study of 160 diabetic kidney patients suggested that Modified Shenqi Dihuang decoction has good curative effect on reducing proteinuria, protecting renal function, restoring the balance of intestinal flora by increasing the numbers of *Bacteroides*, *Bifidobacteria*, and *Lactobacillus*, but decreasing the numbers of Enterobacter, *Enterococcus*, and yeast (Du et al., 2021b). Zicuiyin decoction, which had better efficacy in improving and protecting kidney function in diabetic kidney disease (DKD) patients, could decline eGFR and ameliorate gut microbiota dysbiosis in a multicenter, parallel-control, open-label, randomized clinical trial (Liu et al., 2022). Fushen Granule (FSG) ameliorated BUN and Scr and improved albumin (ALB) by enriching beneficial bacteria associated with metabolism in patients with peritoneal dialysis-related peritonitis (PDRP) in a randomized controlled trial (Lin W. et al., 2021). Supplementation of amylose resistant starch HAM-RS2 (20 g/d resistant starch) led to an improvement of constipation severity and renal function, elevation in *Faecalibacterium*, and a decrease in serum urea, IL-6, TNF-α, and Malondialdehyde (MDA) of systemic inflammation in hemodialysis (HD) patients (Tayebi Khosroshahi et al., 2018; Laffin et al., 2019; Jia et al., 2021). Long-term synbiotic supplementation for 12 months, consisted of high-resistant starch fiber supplement HI-MAIZE 260 (20 g/d, 50% resistant starch) and the probiotic components of nine strains from *Bifidobacteria*, *Lactobacillus*, and *Streptococcus* genera, could reduce eGFR and increase Scr concentration by altering the gut microbiome with an enrichment of *Bifidobacterium* and *Blautia* spp. in stage 3–4 CKD patients (McFarlane et al., 2021). While short-term treatment of synbiotic (Nutrihealth®), containing a mix of probiotics and a prebiotic fiber, omega-3 fatty acids, and vitamins, could increase *Bifidobacterium* counts and maintain the intestinal microbial balance in Mexican patients with ESRD(Cruz-Mora et al., 2014).

The second benefit of supplementation with natural products is uremic toxin removal in the clinical application of CKD treatment. The curcumin supplementation showed a significant decrease in PCS plasma levels in HD patients (Salarolli et al., 2021) and reduced the inflammatory response by modifying the gut microbiota structure of CKD patients after 3-month administration (Pivari et al., 2022). In a clinical trial of 60 peritoneal dialysis patients, Shen-Shuai-Ning granules could decrease IS serum concentration after 12 weeks of treatment (Chen et al., 2018). Supplementation with the functional food HI-MAIZE^®^ 260 cookies (16 g/d resistant starch) could increase fiber intake, reduce IL-6 and IS plasma levels (Esgalhado et al., 2018), decrease different growth factors (De Paiva et al., 2020), and alter the SCFA-producing microbiota in HD patients (Kemp et al., 2021), but no indole-3-acetic acid (IAA) levels or aryl hydrocarbon receptor (AhR) expression in patients with end-stage CKD (Azevedo et al., 2020). However, the unripe banana flour (21 g/d, 48% resistant starch) only decreased IS in the subgroup of participants undergoing peritoneal dialysis (PD) (De Andrade et al., 2021). The prebiotic fructo-oligosaccharide (FOS) decreased the serum total ΔPCS and serum-free Δ%PCS, but not IS and IAA in non-diabetic- and non-dialysis-dependent CKD patients (Ramos et al., 2019). The prebiotic inulin-type fructans restricted the increase in gut-derived indole in PD patients (Li L. et al., 2020). The ß-glucan prebiotic (50% ß-glucan) significantly altered the levels of total and free p-cresyl glucuronide (pCG) and lowered the beta diversity of the gut microbiome in stage 3–5 CKD predialysis participants (Ebrahim et al., 2022). The prebiotic oligofructose-inulin significantly increased intraparticipant microbiome diversity and reduced serum PCS concentrations in HD patients (Meijers et al., 2010; Raj et al., 2021). Inulin with low-protein diet modified gut microbiota and reduced inflammatory factors (TNF-α and NOX2) and metabolic parameters (serum uric acid (SUA) and C-reactive protein (CRP)) in the plasma of CKD patients (Lai et al., 2019). A synbiotic Probinul-Neutro lowered total plasma PC concentrations in non-dialyzed CKD patients with stage 3–4 CKD (Guida et al., 2014). Two-month administration of the synbiotic NatuREN G^®^ resulted in the amelioration of the abdominal pain and constipation symptoms and a decrease in free IS in the stage 3b–4 CKD group (Cosola et al., 2021). The synbiotic capsules (GeriLact brand, Iran) supplementation containing a mix of probiotics and FOS as prebiotic might increase IS and parathyroid hormone levels in HD patients (Mirzaeian et al., 2020). A low-protein diet plus a new formulation of probiotics (*Bifidobacterium longum* and *Lactobacillus reuteri*) was effective in reducing IS in patients affected by advanced CKD (De Mauri et al., 2022). Synbiotic therapy, which consisted of a combination of high-molecular weight inulin, FOS, and galacto-oligosaccharides (GOSs) and the probiotic component including nine strains from the *Lactobacillus*, *Bifidobacteria*, and *Streptococcus* genera, decreased serum PCS of CKD patients with enrichment of *Bifidobacterium* and depletion of Ruminococcaceae after 6 weeks (4-week washout) in a randomized, double-blind, placebo-controlled, crossover trial (Rossi et al., 2016). However, supplementation with trans-resveratrol (Alvarenga et al., 2022) and cranberry dry extract (Teixeira et al., 2022) did not reduce the plasma levels of uremic toxins IS, PCS, and IAA produced by the intestinal microflora in patients with stage 3–4 CKD. In brief, natural products from plants and microorganisms may be a promising treatment for CKD patients in clinical practices.

## 4 Discussion

The studies summarized in this review provide correlative insights into how host–gut microbiota–natural products from plant and microorganism interactions can contribute to CKD management. Advances in constantly updated sequencing technologies let investigators determine the change in microorganisms in experiments with CKD animals treated with different traditional Chinese formulas, crude extracts, natural polymers, and phytochemicals (Table 1). Abnormal changes in the gut microbiota of CKD animals make the pathogenic bacteria-producing urinary toxin accumulate in the blood and fail to be eliminated by the impaired kidney. Simultaneously, the damage to the intestinal epithelial barrier increases the permeability of the intestinal mucosa, which allows pathogenic bacteria and enterogenous endotoxins to translocate into the blood circulation and activates the immune system of the intestinal mucosa. All of these contribute to the imbalance in intestinal microecology, the inducement of systemic inflammatory response, and the impairment of kidney tissue and function. The aforementioned intestinal events are not mutually exclusive. Natural products from plants and microorganisms may act on the dysbiosis of intestinal microecology in CKD through a variety of channels; for example, a natural polysaccharide may modulate the structure of gut microflora, fortify the intestinal mucosa barrier, and reduce the inflammatory reaction in the colon tissue. Even components from the same herb may have different targets. For instance, ethanol extract from *S*. *miltiorrhiza* could increase the diversity of intestinal microflora and regulate the amount of SCFAs, which display the multi-target characteristics of natural products. Interestingly, herbal medicine plus probiotics or synbiotics appeared to strengthen their effectiveness on the dysbiosis of gut microbiota.

Previous studies on the human microbiome have identified correlative microorganisms associated with CKD patients. Transferring the CKD patients’ microbiome into germ-free mice aggravated CKD-associated phenotypes, which proved the causality between gut microflora and the pathogenesis of CKD (Wang X. et al., 2020). Gut microflora is thought to be the most potential therapeutic target for CKD treatment. Accumulating studies have demonstrated that an ancient classic herbal formula, natural polysaccharide, or bioactive compound could alter the diversity of the intestinal microbiota and the production of uremic toxins in CKD patients (Table 2). However, different kinds of natural active products from plants and microorganisms have different mechanisms and limits that need to be distinguished, though they are similar adjuvant treatments *via* targeting gut microbiota. Fermentable dietary fibers, such as inulin, FOS, galacto-oligosaccharides (GOSs), and resistant starch, are utilized as prebiotics that are selectively digested by host microorganisms, which could stimulate the growth of one or a limited number of beneficial bacteria and reduce the uremic toxin released in the colon. However, common side effects including mild diarrhea and gassiness were often accompanied with administration of these fibers (Sanctuary et al., 2019); Moreover, FOS and GOS were reported to have harmful effects on the glucose metabolism by reducing butyrate-producing microbes (Liu et al., 2017). Therefore, whether they have potential adverse effects and became intolerant, especially in long-term interventions, need to be further researched. The administration of probiotic supplementation only failed to reduce uremic toxins and inflammatory markers in CKD patients (Borges et al., 2018), and the combination of probiotics with healthy components like prebiotics and bioactive compounds, called synbiotics, could benefit the gut ecosystem and host life. Many cohort studies of synbiotics listed in the review are short-term RCTs with small-scale participants, and we are unable to rule out the bioactive components or a specific bacterium that mainly contribute to the efficacy, not to mention the best dosage and proportion of prebiotics, route of administration, and duration of intervention. The variation of a single element in the study may result in different outcomes.

Herbal medicine has been one of the most widely used alternative methods for the prevention and treatment of CKD in China and other Asian countries for hundreds of years. Our review demonstrated that different proportions of traditional herbal formulas or different herbal ingredients have different focuses on the intestinal dysbacteriosis and abnormal immune system of CKD, which presented the great advantage of herbal medicine in CKD treatment. The synergistic effects of multiple ingredients of herbal medicine on the gut microbiota of patients with CKD are reflected in two aspects: on the one hand, the bioactive ingredients have multi-target characteristics. The discrepancy in the regulatory objectives of gut microbiota makes them supplement each other. Taking Astragali Radix as an example, where *Astragalus* polysaccharide and astragaloside IV are the most important bioactive ingredients purified from Astragali Radix, both of which have to be biotransformed by gut microbiota to get better potency (Wang and Chen, 2017; Hong et al., 2021). *Astragalus* polysaccharide can modify the gut flora structure and fortify the intestinal mucosa barrier of the colon tissue, while astragaloside IV can protect against kidney injury by alleviating oxidative stress, attenuating mitochondrial dysfunction, and reducing inflammatory reactions in the kidney tissue (Zhang et al., 2020). On the other hand, the different proportions or bioactive ingredients cooperate with each other to improve the therapeutic effects by regulating the structure and metabolites of gut microbiota. Moreover, the synergistic effect can also reduce the possible toxic and side effects during the treatment process. Taking RR and CF as examples, they have been used together to treat CKD for thousands of years. The relative abundance of probiotic genera *Ruminococcaceae* and *Lachnospiraceae* was decreased in CKD rats, which could not be significantly recovered by the treatment of RR or CF, respectively, but they are notably increased in the RR + CF treatment groups (Zhang et al., 2021d). However, there are still some problems that need to be solved. First of all, even though many bioactive components from herbal medicine were identified through modern technology including standardized phytochemistry, pharmacology, pharmacokinetics, pharmacodynamics, and toxicology research procedures (Lin T. L. et al., 2021), the bottleneck barrier of herbal medicine development i.e., investigating how the complex ingredients in herbal medicines work together in a synergistic pattern is still not fully elucidated. Second, herbal medicine can avoid the adverse therapeutic effect caused by Western medicine treatment in the clinical practices of CKD treatment (Zhao et al., 2021), but most studies of these herbal medicines are focused on animal experiments. There are few clinical trials about herbal medicine plus first-line Western medicine for CKD treatment except for the multicenter clinical trial of *Abelmoschus manihot* and irbesartan (Zhao et al., 2022). High-quality interventional trials of well-studied, high-quality RCTs investigating herbal medicine treatment in CKD are still lacking. Third, though a variety of active substances identified from the herbal medicines, such as alkaloids, polysaccharides, glycosides, lipids, and vitamins, provide material basics for the multi-level and multi-target characteristic of herbal medicine, side effects and safety validations of herbal medicine on CKD patients need further large size and long duration clinical trials.

Another important aspect that has to be considered is that the ingredients of natural products may be metabolized by gut microflora and affect their structures and efficacy. Accumulating evidence showed that the bioactive compounds from herbal medicines like Astragali Radix and *S*. *miltiorrhiza* were effective in ameliorating kidney injury *via* gut microbiota biotransformation (Wang and Chen, 2017; Cai et al., 2019). It was reported that APS could be fermented by intestinal microorganisms *Desulfovibrio vulgaris* to produce acetic acids (Hong et al., 2021). Astragaloside IV (ASIV) is the most abundant saponin purified from Astragali Radix. It is gradually deglycosylated by human intestinal flora and transformed into cycloastragenol (Wang and Chen, 2017), which is the effective form with better permeability and absorptivity to improve the bioavailability *in vivo* (Zhai et al., 2016). Tanshinones and salvianolic acid from *S*. *miltiorrhiza* would exert effect after being metabolized through methylation, demethylation, dehydrogenation, hydrogenation, and hydroxylation by intestinal bacteria (Cai et al., 2019).

Identifying gut microbiota as the alternative target of natural products from plants and microorganisms in the treatment of CKD still face more challenges. These associative studies should spur further causal investigations by using modern microbial technology, such as genomic sequencing, germ-free (GF) animals, and antibiotic treatments. Additionally, to understand how natural products from plants and microorganisms affect the gut microbiota in CKD patients and to develop new alternative therapies for CKD treatment, the strain- and molecular-level connections between the gut microbiome, natural products from plants and microorganisms, and host CKD phenotype should be established in future research based on the hypotheses developed from these correlative studies (Chaudhari et al., 2021). The systematic research on functional compounds from natural products metabolized by gut microbiota might seek out a new approach to uncover these problems. Identification and functional testing of gut microbiota metabolites related to natural product components would deepen the understanding of how the natural products modulate CKD patients’ physiology *via* targeting gut microbiota by using cutting-edge omics platforms including next-generation sequencing (NGS), proteomics, metabolomics, and cultureomics. These strategies have pushed the development of novel disease-related probiotics, prebiotics, and functional proteins for the treatment of CKD by targeting the dysbiosis of the gut microbiota in recent years (Lobel et al., 2020; Zhu et al., 2021). The synergistic effect of multiple methods such as herbal medicine and these disease-related probiotics or prebiotics would provide a novel therapeutic method for CKD patients.

## 5 Conclusion

In summary, the literature analyzed in this review suggests a great advantage in the adoption of natural products from plants and microorganisms to treat CKD *via* targeting gut microbiota. These natural products from plants and microorganisms have an impact on the biogenesis and progression of CKD and its relative metabolic complications through alteration of the diversity and composition of the gut microbiota. More research should be carried out to prove the causal role of intestinal microflora in the treatment of CKD by natural products from plants and microorganisms based on these associative studies.
